# Severe hypoxemia: which strategy to choose

**DOI:** 10.1186/s13054-016-1304-7

**Published:** 2016-06-03

**Authors:** Davide Chiumello, Matteo Brioni

**Affiliations:** Dipartimento di Anestesia, Rianimazione ed Emergenza-Urgenza, Fondazione IRCCS Ca’ Granda—Ospedale Maggiore Policlinico, Via F. Sforza 35, Milan, Italy; Dipartimento di Fisiopatologia Medico-Chirurgica e dei Trapianti, Università degli Studi di Milano, Milan, Italy

## Abstract

**Background:**

Acute respiratory distress syndrome (ARDS) is characterized by a noncardiogenic pulmonary edema with bilateral chest X-ray opacities and reduction in lung compliance, and the hallmark of the syndrome is hypoxemia refractory to oxygen therapy. Severe hypoxemia (PaO_2_/FiO_2_ < 100 mmHg), which defines severe ARDS, can be found in 20–30 % of the patients and is associated with the highest mortality rate. Although the standard supportive treatment remains mechanical ventilation (noninvasive and invasive), possible adjuvant therapies can be considered. We performed an up-to-date clinical review of the possible available strategies for ARDS patients with severe hypoxemia.

**Main results:**

In summary, in moderate-to-severe ARDS or in the presence of other organ failure, noninvasive ventilatory support presents a high risk of failure: in those cases the risk/benefit of delayed mechanical ventilation should be evaluated carefully. Tailoring mechanical ventilation to the individual patient is fundamental to reduce the risk of ventilation-induced lung injury (VILI): it is mandatory to apply a low tidal volume, while the optimal level of positive end-expiratory pressure should be selected after a stratification of the severity of the disease, also taking into account lung recruitability; monitoring transpulmonary pressure or airway driving pressure can help to avoid lung overstress. Targeting oxygenation of 88–92 % and tolerating a moderate level of hypercapnia are a safe choice. Neuromuscular blocking agents (NMBAs) are useful to maintain patient–ventilation synchrony in the first hours; prone positioning improves oxygenation in most cases and promotes a more homogeneous distribution of ventilation, reducing the risk of VILI; both treatments, also in combination, are associated with an improvement in outcome if applied in the acute phase in the most severe cases. The use of extracorporeal membrane oxygenation (ECMO) in severe ARDS is increasing worldwide, but because of a lack of randomized trials is still considered a rescue therapy.

**Conclusion:**

Severe ARDS patients should receive a holistic framework of respiratory and hemodynamic support aimed to ensure adequate gas exchange while minimizing the risk of VILI, by promoting lung recruitment and setting protective mechanical ventilation. In the most severe cases, NMBAs, prone positioning, and ECMO should be considered.

## Background

Since the first description, acute respiratory distress syndrome (ARDS) has been redefined several times in order to ameliorate the accuracy of the clinical diagnosis [1–3]. However, independently from the different proposed definitions, the hallmark of ARDS is the arterial hypoxemia refractory to the oxygen therapy, due to pulmonary shunt. Two thresholds for severe hypoxemia (PaO_2_/FiO_2_ < 150 or 100 mmHg) have been proposed; both of these are associated with the highest mortality (up to 45 %), duration of mechanical ventilation, and risk of ventilation-induced lung injury (VILI) [3–5].

According to the recent Berlin definition, ARDS is characterized by an inflammatory lung edema of recent onset, causing severe respiratory failure which requires invasive ventilation or noninvasive ventilation (NIV) [3]. Classically, the increases in lung edema (i.e., lung weight) and in pleural pressure, raising the hydrostatic pressure transmitted throughout the lung, reduce the lung gas volume and promote the development of nonaerated regions (consolidated or atelectatic), mainly in the more dependent lung regions [6].

The increasing knowledge of ARDS pathophysiology through the years has led to suggestions for the application of a lung-protective ventilatory strategy, which in addition to ensuring adequate oxygenation (PaO_2_ between 60 and 80 mmHg) should minimize VILI [3]. Unfortunately, completely “safe” lung-protective ventilation does not exist, and the ventilatory support should be individualized according to the best compromise among respiratory mechanics, recruitability, gas exchange, and hemodynamics.

In this clinical review we will present expert opinion on the different lung support and adjuvant therapies which have been proposed within the framework of the clinical management of ARDS with severe hypoxemia (i.e., severe ARDS, with PaO_2_/FiO_2_ < 100 mmHg).

### Noninvasive support

The possible use of NIV in patients with ARDS, although it could reduce the intrapulmonary shunt and decrease the work of breathing, is still controversial because of the high risk of failure and the possible risks associated with a delay in starting invasive mechanical ventilation. The last consensus conference on NIV pointed out that “larger controlled studies are required to determine the potential benefit of adding NIV to standard medical treatment in the avoidance of endotracheal intubation” [7]. In a recent meta-analysis, which included 13 studies with a total of 540 patients mainly treated with bilevel positive airway pressure, the intubation rate ranged between 30 and 86 % and the mortality rate from 15 to 71 % [8]. Unfortunately, the majority of these studies were not randomized, the studies presented great heterogeneity, and none of them compared NIV with invasive ventilation; consequently, it is not possible to make firm conclusions. Because of the high risk of failure, NIV should be reserved for ARDS patients without extralung organ failures, and should be provided in the ICU where strict monitoring and prompt intubation is always possible without delay. If after the first few hours there is no significant improvement in gas exchange or the respiratory rate, NIV should be stopped and invasive mechanical ventilation should be started.

A possible alternative to NIV could be application of the high-flow nasal cannula (HFNC) system, which can deliver a very high, heated, and humidified oxygen flow through the nose [9]. HFNCs are able to increase the end-expiratory lung volume, reduce the work of breathing, and improve CO_2_ clearance and oxygenation. In addition to these beneficial effects, and contrary to NIV, HFNCs do not require any nasal or mask interface, which significantly improves long-term tolerance and use. HFNCs, originally developed for neonatal and pediatric settings, have been evaluated recently in adult patients. In an observational study in ARDS patients (33 % and 29 % with severe and moderate ARDS respectively), HFNCs failed in 40 % of the patients, who were subsequently intubated [10]. The main reasons for intubation included worsening of hypoxemia and hemodynamic or neurologic failure [10]. This rate of intubation was similar to that found by Antonelli et al. (46 %), who tested NIV in ARDS patients [11]. Presently, only one randomized study in patients with acute respiratory failure without cardiogenic edema has evaluated HFNCs (with gas flow rate of 50 l/minute) compared with NIV (set with a pressure support to ensure a tidal volume between 7 and 10 ml/kg and positive end-expiratory pressure (PEEP) between 2 and 10 cmH_2_O) and oxygen therapy [12]. The intubation rate was not different between the three groups (from 38 to 50 %), but the intensive care mortality was significantly lower in the HFNC group.

Presently the indications and the standards of monitoring for HFNCs in ARDS patients are similar to those of NIV.

### PEEP and lung recruitment

Although PEEP and lung recruitment are usually considered separately in the lung-protective ventilation protocols, they are strictly related. Accordingly to a physical model, in order to recruit the lung (i.e., to inflate the collapsed lung regions) and to keep these regions open, we have to overcome the superimposed pressure generated by the lung mass and by the chest wall [13]. To recruit the lung, several types of recruitment maneuvers (RMs) have been proposed: the sigh, in which higher tidal volumes are intermittently delivered during ventilation; the sustained inflation, induced by a static increase in airway pressure applied for 20–40 seconds; and the extended sigh, in which a stepwise increase of PEEP is applied [14]. Independently of the applied RMs, the main goal is to reinflate the “closed” pulmonary units by applying a high transpulmonary pressure for an adequate period of time. In the majority of patients, a RM is able to improve oxygenation for a certain period of time without major side effects [14]; however, the RMs alone were not associated with a reduction in mortality [15].

**Fig. 1:**
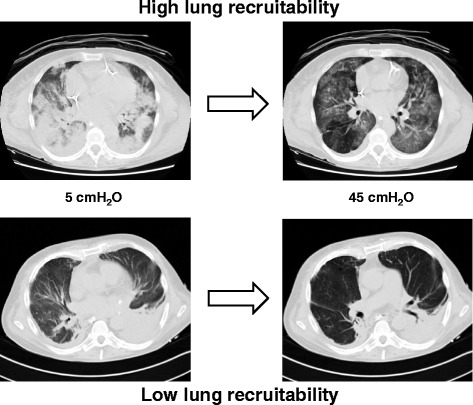
Example of lung CT scan of patients with high (*upper panel*) or low (*lower panel*) potential of lung recruitment

During the decades, the “philosophy” of PEEP has changed significantly. From a simple tool used to increase oxygenation at the beginning of the history of mechanical ventilation, PEEP has in recent years gained a primary role in the framework of the lung-protective strategy, avoiding intratidal opening and closing and decreasing lung inhomogeneities [4, 16–18]. Owing to the different amounts of lung edema, the total lung recruitability (estimated by lung computed tomography (CT) scan) was found to range from 0 to 70 % of the total lung weight [19] (Fig. 1). Presently, although the lung CT scan requires the transport of patients outside the ICU and the use of X-ray radiation, it remains the gold standard to compute lung recruitability [20, 21]. The use of a visual scale to estimate lung recruitment and the application of a low-dose protocol for CT scan acquisition have shown promising results [22, 23]. In addition, a recent observational study showed that the CT scan, independent from estimation of lung recruitment, contributed to diagnosis in 53 % of patients and induced a therapeutic change in 54 % of cases [24]. As an alternative, lung ultrasound showed a reliable accuracy in estimating lung recruitability, but further studies are necessary to confirm its use [25].

Although several experimental and observational studies found a beneficial effect for the use of higher PEEP in ARDS [19, 26, 27], the three most recent randomized trials (ALVEOLI, ExPress, and LOV) did not show any difference in outcome between a low and a high PEEP ventilator strategy [28–30]. However, when combining these data considering only the subgroup of the most severe patients (PaO_2_/FiO_2_ < 200 mmHg), the use of higher PEEP level significantly decreased mortality [31, 32]. This suggests that the greater the severity (and the higher the amount of lung edema), the higher the positive effect of PEEP in reducing VILI. This has also been confirmed in an observational study, in which higher PEEP levels significantly reduced the opening and closing effects only in patients with higher recruitability [16]. However, the relationship between lung edema/mass and recruitability has been questioned by Cressoni et al. [13], who found that the PEEP levels necessary to keep the lung open are independent from total lung recruitability. These results suggest that recruitability depends also on the nature of edema, time of onset, and distribution of the disease within the lung parenchyma.

Several approaches have been proposed to tailor PEEP for the individual patient. The most common approach is to titrate PEEP according to an oxygenation/saturation target based on a PEEP/FiO_2_ table [30]. An alternative method, based on respiratory mechanics, is to increase PEEP by maintaining a constant tidal volume, not overcoming a safe limit of airway pressure (26–28 cmH_2_O) [28], or, after a RM, to decrease PEEP until a reduction of compliance appears [33, 34]. Despite the possible uncertainties regarding the end-expiratory absolute esophageal pressure as a reliable estimation of the pleural pressure [35], Talmor et al. [36] showed a better oxygenation and compliance when PEEP was set according to an end-expiratory transpulmonary pressure between 0 and 10 cmH_2_O (absolute method). Alternative to the absolute value, the changes in esophageal pressure due to PEEP and tidal volume (elastance method) have been used to compute the total end-inspiratory transpulmonary pressure, as a better marker of lung stress compared with airway inspiratory pressure in the presence of alteration in chest wall elastance [37]. By computing the end-inspiratory transpulmonary pressure vs airway pressure, Grasso et al. [38] showed that it was possible to increase PEEP, improving oxygenation and avoiding extracorporeal membrane oxygenation (ECMO) support without overcoming the lung stress. However, when these two methods (absolute and elastance) were compared, the resulting PEEP levels were significantly discordant and furthermore the recommended PEEP changes were in the opposite direction in up to 30 % of the patients [39].

Recently our group compared the previous published methods for selecting PEEP (based on gas exchange, respiratory mechanics, and transpulmonary pressure) with lung recruitability and severity of the disease [40]. The method based on gas exchange (i.e., FiO_2_/PEEP table of the LOV study [30]) was the only one which provided PEEP levels according to the severity of the disease; on the contrary, the other methods suggested similar levels of PEEP that were not related to the severity or to lung recruitability [40]. Interestingly, obese patients with ARDS presented a significantly lower lung gas volume but similar lung recruitability and chest wall elastance compared with normal body weight patients [41].

Based on the available data, it is clear that “perfect PEEP”—which can simultaneously provide the best oxygenation, compliance, and reduction of VILI—does not exist. Therefore, we suggest performing, in the acute phase, a stratification of patients according to the severity of ARDS before any PEEP selection. This can be done easily by ventilating the patient with pure oxygen at PEEP 5 cmH_2_O [5]. In the case of severe ARDS, the lung recruitability should be computed by lung CT scan or lung ultrasound, and high PEEP levels (i.e., >15 cmH_2_O) following the PEEP/FiO_2_ table of the LOV study [30] should be applied. On the contrary, in mild to moderate ARDS, low PEEP levels (<10 cmH_2_O) can be applied safely.

Improvement in oxygenation can be due simply to a hemodynamic effect (reduction of cardiac output and right-to-left shunt) without any effect on lung recruitment. Thus, before any PEEP trial the patients should present hemodynamic stability, and any changes in hemodynamics during the trial should be evaluated. In addition, to avoid lung overstress, the transpulmonary pressure should be measured while simultaneously titrating PEEP and the tidal volume.

### Tidal volume

One of the main determinants of VILI is the generation of an unphysiologic stress (tension) and strain (deformation), which depends both on the size of the delivered tidal volume and the amount of lung resting volume [42]. The lower the tidal volume and/or the higher the resting volume, the lower will be the generated stress/strain, which is associated with lung damage irrespective of hypercapnia [37, 43]. Basing on these findings, a seminal study (the ARMA trial) showed a reduction of 22 % in mortality using a tidal volume of 6 ml/kg of ideal body weight compared with 12 ml/kg [44]. A recent Cochrane Collaboration meta-analysis considering the randomized controlled trials which compared ventilation using either a lower tidal volume or lower airway pressure (i.e., plateau pressure ≤ 30 cmH2O) with a ventilation using a higher tidal volume clearly showed that mortality at day 28 was significantly reduced by lung-protective ventilation [45]. The authors thus concluded that ventilation with lower tidal volumes should become a routine strategy treatment for ARDS, stopping the investigators from carrying out additional trials. However, despite almost two decades since the publication of the ARMA trial, the low tidal volume is still not used routinely [46], although it has also been proved that this strategy is clinically safe without the need for an increase in the dosage of sedative or neuromuscular blockers [47, 48].

Usually tidal volume is selected according to ideal body weight; however, ideal body weight is poorly related to the resting volume, and a similar tidal volume can generate different lung stress/strain in different patients with the same anthropometric characteristics [37]. Recently, the use of airway driving pressure has been proposed to better individualize the tidal volume [49]. The airway driving pressure, measured as the ratio between the tidal volume and respiratory system compliance, should better reflect lung stress/strain because the respiratory system compliance is related to the available lung gas volume [50, 51]. Recently, Amato et al. [49] found that the airway driving pressure, in different combinations of tidal volume and levels of PEEP, was the strongest factor associated with outcome in ARDS patients. The airway driving pressure could thus be a useful tool to identify patients at risk of VILI.

### Modality of mechanical ventilation

Presently the two most commonly used modes of mechanical ventilation are pressure-controlled ventilation (PCV) and volume-controlled ventilation (VCV). With PCV the delivered volume changes according to the characteristics of the respiratory system, and the inspiratory flow presents a decelerating shape; in VCV the delivered volume remains constant, while the airway pressure is variable and the inspiratory flow has a constant shape. It has been hypothesized that PCV could present higher benefits in reducing VILI, due to decelerating inspiratory flow and the changes in delivered tidal volume according to the patient’s disease. In order to assess any possible advantage of PCV compared with VCV, Chacko et al. [52] performed a systematic review and meta-analysis without showing any difference in mortality or risk of barotrauma between the two modes.

Compared with controlled mechanical ventilation, assisted mechanical ventilation could provide some beneficial effects by reducing the level of sedation, maintaining the respiratory muscle activity, and promoting a more homogeneous distribution of the ventilation [53]. In a small cross-over study of moderate ARDS, similar tidal volumes and lung distending pressure were found among pressure support ventilation (PSV), PCV, and neurally adjusted ventilatory assist (NAVA) [54]. When not properly applied, however, the use of assisted mechanical ventilation in severe ARDS, similarly to NIV, could negatively affect the outcome and increase VILI by increasing the transpulmonary pressure, work of breathing, and rapid shallow breathing [53]. Further studies are required to clarify the role of PSV and NAVA in severe ARDS.

### Oxygenation and CO_2_ target

The commonly recommended oxygenation target ranges between 88 and 95 %. However, in clinical practice a more liberal approach aimed to maintain an arterial saturation higher than 96 % is often used, due to physician perception of higher patient safety. To better understand the possible benefits of a more liberal strategy, Panwar et al. [55] randomized ARDS patients to receive an arterial saturation higher than 96 % or between 88 and 92 %. The liberal strategy did not influence both the number of organ failures and the outcome.

The application of a low tidal volume ventilation strategy can result in hypercapnia, but does not present major side effects and is well tolerated. However, hypercapnia can increase a patient’s respiratory drive, and was independently related to the administration of neuromuscular blocking agents (NMBAs) [56]. Although the optimal CO_2_ level is still unclear, in the absence of right cardiac failure and raised intracranial pressure up to 70 mmHg of arterial CO_2_ with a pH of 7.20 has been found to be safe [57, 58].

### Neuromuscular blocking agents

NMBAs are frequently used to abolish the inspiratory and expiratory efforts of patients, in order to improve patient–ventilator synchrony and to minimize the muscle oxygen consumption [59]. In addition, NMBAs can reduce the stress/strain generated in the lung by reducing the negative increase in pleural pressure during spontaneous efforts [59]. However, NMBAs can increase the risk of ICU-acquired weakness and diaphragmatic dysfunction, prolonging the duration of mechanical ventilation. In the last multicenter randomized trials evaluating PEEP, NMBAs were given in up to one-half of the enrolled patients [28, 30]. Although NMBAs were applied without a predefined protocol, they were more frequently used in patients with a higher APACHE III score and hypoxemia, without any difference between the low and high PEEP groups [56]. To clarify the role of NMBAs in ARDS, several trials have been published in recent years. In 2004 Gainnier et al. [60] evaluated, in severe ARDS patients, the effects of 48 hours of NMBA infusion added to a deep sedation (level 6 accordingly to the Ramsay scale). At 48, 96 and 120 hours the NMBA group had higher oxygenation without any difference in the airway plateau pressure, PEEP levels, and amount of drugs used for sedation. A further small randomized trial showed that a continuous infusion of NMBAs during the first 48 hours caused a significantly lower local (pulmonary) and systemic (blood) inflammatory response (IL-6 and IL-8), lower PEEP levels, and better oxygenation [61]. In the largest multicenter trial, 340 severe ARDS patients were randomized to receive a 48-hour continuous infusion of NMBAs or a placebo [62]. The NMBA group showed a significant reduction in the 90-day mortality rate, with a higher amount of ventilator-free days and lower incidence of pneumothorax. The incidence of ICU-acquired paresis at intensive care discharge was not different. A subsequent meta-analysis, considering the randomized controlled trials of adult patients with ARDS randomized to receive NMBAs, found that the patients treated with NMBAs presented a lower mortality (risk ratio 0.71 (95 % confidence interval 0.55–0.90)) and fewer days of mechanical ventilation, with a higher number of ventilator-free days at day 28 (*p* = 0.0020) and lower episodes of barotrauma (*p* = 0.030) [63].

Based on the available data, NMBAs should be reserved for the most severe ARDS patients, mainly in the acute phase and in the first hours of mechanical ventilation, to ensure adequate patient–ventilator synchrony, avoiding the generation of a harmful transpulmonary pressure. However, the adjustment of the ventilator setting and the need for paralysis should, at least, be evaluated every day.

### Prone positioning

More than 30 years ago several observational studies reported that prone positioning was able to increase arterial oxygenation in the majority of patients with acute respiratory failure [64, 65]. Prone positioning was thus reserved as a rescue treatment in case of life-threatening hypoxemia. The main consequences of prone positioning, which can be all or in part present, include a redistribution of the lung densities with a recruitment of the dorsal regions, an increase in the chest wall elastance, a reduction in the alveolar shunt, and a better ventilation/perfusion ratio with improvement in oxygenation and CO_2_ clearance, a more homogeneous distribution of ventilation with a reduction of VILI, and a reverse of right heart failure [66, 67]. Based on these favorable effects, several trials have been planned since 1996 to test prone positioning in ARDS patients. The first studies, enrolling patients with moderate to severe ARDS without applying protective mechanical ventilation, did not show any beneficial effect from use of short-term prone positioning (<8 hours per day) [68, 69]. The subsequent two trials, which enrolled more severe hypoxemic patients with a longer period of prone positioning (20 hours per day), also did not show any beneficial effects [70, 71]; however, a meta-analysis of the previous studies suggested a significant survival benefit for patients with PaO_2_/FiO_2_ < 140 mmHg at admission [72]. With this background, Guerin et al. [73] conducted a multicenter randomized study on the use of long-term prone positioning (at least 16 hours per day) in severe ARDS. The PEEP was selected from a PEEP/FiO_2_ table of the low PEEP arm of the ALVEOLI study [29] and the tidal volume was strictly controlled to 6 ml/kg of ideal body weight. The mortality at 28 days was significantly lower in the prone positioning group (16 % vs 32 %), the rate of successful extubation was higher, and the mean duration of prone positioning was 17 ± 3 hours [73].

Owing to the reduction of the harmful effects of mechanical ventilation both by prone positioning and NMBAs when evaluated separately, adding NMBAs in prone positioning could have a synergistic effect in improving the oxygenation, decreasing the duration of mechanical ventilation, and improving the final outcome [74].

According to the recent Berlin definition, prone positioning should be reserved for severe ARDS patients, especially in the acute phase in which the amount of edema, atelectasis, and lung recruitability is higher, and for a longer period of time [4]. Although in a minority of the patients the oxygenation does not improve, prone positioning has additional beneficial effects, mainly related to the reduction of VILI, to be considered. Although prone positioning presents some technical issues, when it is performed by a skilled team the adverse affects are relatively low and they are significantly overcome by the beneficial effects. However, before any change in the position of critically ill patients, the presence of any absolute contraindication (e.g., pregnancy, unstable fractures, open abdominal wounds, extreme hemodynamic instability) has to be considered [66].

### Extracorporeal membrane oxygenation

Standard ECMO support is commonly performed by a veno-venous access in which the blood is drained by the superior or inferior vena cava and reinfused in the right atrium. The artificial lung is able to provide adequate blood CO_2_ removal and oxygenation, allowing reduction of mechanical ventilation (tidal volume, respiratory rate, and oxygen fraction) and minimization of VILI. The first applications of ECMO in patients with acute respiratory failure did not show any benefit; some years later, starting in 1985, several studies found a decrease in the mortality rate, ranging between 21 and 50 % [75]. However, only one randomized trial comparing ECMO with standard care have been performed recently (the CESAR trial) [76]. In this trial, patients with ARDS were referred to a single center and managed with ECMO or treated with conventional mechanical ventilation. Mechanical ventilation during ECMO provided lung rest with a peak inspiratory pressure of 20–25 cmH_2_O and PEEP between 10 and 15 cmH_2_O. At 6 months the ECMO group presented a higher survival rate compared with the control group (63 % vs 47 %; *p* = 0.03), while the quality of life and spirometric parameters were not different. Despite these positive results, the CESAR trial has been criticized because ventilator treatment in the control group was not standardized, 30 % of the patients were not ventilated with a lung-protective strategy, and all patients requiring ECMO were allocated only in one skilled center. From these data it is thus not possible to conclude that ECMO is superior to mechanical ventilation [75]. Despite the absence of any further randomized trial, but mainly based on the theoretical benefit of lung rest and on several case series, the use of ECMO has continued to increase worldwide. However, some uncertainty remains; as an example, the recent Scandinavian clinical practice guideline on mechanical ventilation in ARDS does not take into account the use of ECMO [77].

To better allocate resource utilization and to help the physician choose the appropriate treatment, because of a lack of recognized criteria for ECMO application, Schmidt et al. [78] in a retrospective analysis identified eight sample clinical variables collected at ECMO admission which showed good accuracy to predict the probability of survival. In a subsequent study the same author, in a larger database registry of 2355 patients treated with ECMO, identified 12 variables which allowed creation of a well-calibrated survival model [79].

It is worthy to remember that VILI is not completely abolished during ECMO treatment: consequently, prone positioning—similar to conventional mechanical ventilation—should be used as an adjuvant tool. At the present time there is a paucity of data regarding the use of prone positioning during ECMO; for example, the ELSO registry (the largest worldwide) did not collect this information (https://www.elso.org/). However, Kimmoun et al. [80] found that prone positioning for 24 hours, performed in ARDS patients during ECMO, significantly increased oxygenation and respiratory system compliance in the majority of patients without major adverse effects.

## Conclusions

**Fig. 2:**
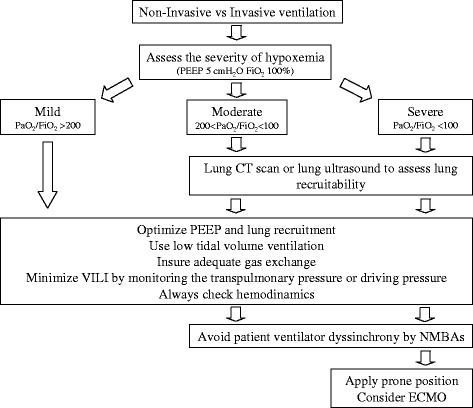
Simple flow chart for a holistic approach to mechanical ventilation in ARDS patients. *CT* computed tomography, *ECMO* extracorporeal membrane oxygenation, *NMBA* neuromuscular blocking agents, *PEEP* positive end-expiratory pressure, *VILI* ventilation-induced lung injury

Severe ARDS patients should receive a holistic framework of respiratory and hemodynamic support ensuring adequate gas exchange and minimizing VILI (Fig. 2): setting an adequate PEEP level (also considering lung recruitability) is of fundamental importance, while avoiding lung overstress by monitoring transpulmonary pressure (or airway driving pressure) and considering in the most severe cases NMBAs, prone positioning, and ECMO (also in combination). The need for NMBAs and prone positioning should be evaluated daily to avoid delaying the waking of the patient and the onset of early mobilization.

### Key messages

- Severe hypoxemia is present in up to 30 % of ARDS patients, and is associated with higher mortality and longer duration of mechanical ventilation.
- Stratification of the patients is important, in order to identify those who might benefit from adjunctive treatments.
- In all patients, a holistic framework of respiratory and hemodynamic support should be considered.

## Abbreviations

ARDS: acute respiratory distress syndrome
CT: computed tomography
ECMO: extracorporeal membrane oxygenation
HFNC: high flow nasal cannula
NAVA: neurally adjusted ventilatory assist
NIV: noninvasive ventilation
NMBA: neuromuscular blocking agent
PCV: pressure-controlled ventilation
PEEP: positive end-expiratory pressure
PSV: pressure support ventilation
RM: recruitment maneuver
VCV: volume-controlled ventilation
VILI: ventilation-induced lung injury
